# Surgical technique of an innovative patient‐specific metal implant for talar osteochondral lesions

**DOI:** 10.1002/jeo2.70085

**Published:** 2025-04-07

**Authors:** Massimiliano Mosca, Silvio Caravelli, Marco Di Ponte, Giammarco Gardini, Emanuele Vocale, Niek C. Van Dijk, Stefano Zaffagnini

**Affiliations:** ^1^ II Clinic of Orthopadedics and Traumatology‐IRCCS Istituto Ortopedico Rizzoli Bologna Italy; ^2^ Ortopedia Bentivoglio‐IRCCS Istituto Ortopedico Rizzoli Bentivoglio Italy; ^3^ Department of Orthopaedic Surgery Amsterdam Medical Center, University of Amsterdam Amsterdam Zuidoost The Netherlands

**Keywords:** ankle, custom‐made implant, osteochondral lesions

## Abstract

**Purpose:**

Treatment strategies for osteochondral defects (OCDs) of the ankle have substantially increased over the last decade. The development of a small metallic implant to fill the defect has led to the second‐generation patient‐specific metal implant (Episealer Talus® Implant) designed based on computed tomography and magnetic resonance imaging images.

**Methods:**

There is a pool of patients falling into the so‐called ‘treatment gap’, a grey zone composed of active patients with symptomatic OCDs in the context of an otherwise healthy joint, or patients with a failed primary treatment. To minimize the risk of perioperative complications, there are a series of tips and tricks that can be considered.

**Results:**

Correct execution of the operative approach, proper positioning of the guides, posterior capsule and deep deltoid ligament release and the use of Hintermann spreader allow a perfect visualization of the OCDs minimizing the risk of iatrogenic lesions. Correct execution of the medial malleolus osteotomy, release of soft tissue, proper triplanar alignment of the custom‐made guide, its strong stabilization during the reaming and the use of vigorous washes minimizes the potential damage on healthy cartilage. Correct sinking of the implant is crucial; the goal is to place the Episealer Talus at least 0.5 mm below the cartilage surface. Filling a large subchondral cyst with the cancellous bone can be useful to provide better stability of the implant.

**Conclusion:**

Episealer Talus for talar OCDs possibly represents an additional tool for surgeons and patients. It is important to avoid mistakes during implant placement.

**Levels of Evidence:**

Level V, expert opinion.

**Abbreviations**
AA: ankle arthrodesis
ATFL: anterior talofibular ligament
CFL: calcaneofibular ligament
DMR: damage marking report
MMOG: medial malleolus osteotomy guide
OCDs: osteochondral defects
TAR: total ankle replacement

## INTRODUCTION

Osteochondral defect (OCD) of the talus usually results from traumatic insult. For lateral talar defects, trauma has been described in 93%–100% and for medial defects in 61%–70% [10,21]. In the United States, approximately two million acute ankle sprains occur annually [24]; among these, the patients who are subsequently undergoing operative repair in about 60% suffer from OCDs [17]. Further, as much as 73% of ankle fracture patients may suffer from osteochondral damage [13]. About 2/3 of symptomatic OCDs are located on the medial shoulder of the talus and 1/3 are located on the lateral shoulder. Not all the patients report a history of ankle injury [9]. Ischaemia, subsequent necrosis and possibly genetics are aetiologic factors in nontraumatic OCDs that are usually described as Osteochondritis Dissecans [16].

Furthermore, OCDs in identical twins and siblings have been described [1,6,25]. The defect is bilateral in 10% of patients [11].

Traumatic OCDs of the ankle can either heal and remain asymptomatic or progress to deep ankle pain on weight‐bearing and possibly formation of subchondral bone cysts. The development of a symptomatic OCDs depends on various factors, including the extent of damage and insufficient repair of the subchondral bone plate. During loading, compressed cartilage forces its water into the subchondral bone, leading to a localized high increased flow and pressure of fluid in the subchondral bone [18]. This can result in local osteolysis and can develop into a subchondral cyst. The pain does not arise from the cartilage but is caused by repetitive high fluid pressure during walking, which results in stimulation of the highly innervated subchondral bone underneath the cartilage defect [18]. Understanding the natural history and development of OCDs is important for the design of logical treatment strategies and the prevention of progressive joint damage.

Treatment strategies for OCDs of the ankle have substantially increased over the last decade. The choice of treatment is based on the type, size and location of the defect and the alignment. Deep and large defects often lead to unsatisfying results with conservative treatment [15]. Other factors to pay attention to are represented by the age of the patient, the aetiology (primary or secondary lesion) and the surgeon's preferences. Various arthroscopic and open procedures are frequently employed, including reattachment of the fragment, local debridement with fragment removal and bone marrow stimulation by microfracture or microdrilling (anterograde or retrograde) for which good results have been reported [26].

The wide range of treatments for OCDs include also more recent techniques such as autologous chondrocyte implantation for hyaline cartilage regeneration, Matrix‐associated autologous chondrocyte transplantation/implantation and osteochondral transplantation (osteochondral autograft transfer system) for cartilage replacement in larger defects or as a salvage procedure. However, these techniques are associated with donor site morbidity or involve two‐stage surgery [22]. Transplantation of so‐called (osteochondral) mega grafts, such as autologous bone grafts or allografts, is used for a very large OCD that cannot be reconstructed otherwise [2]. The last step of treatment is represented by the total ankle replacement (TAR) or ankle arthrodesis (AA) after the failure of all previous treatments.

However, there is a pool of patients falling into the so‐called ‘treatment gap’, a grey zone composed of active patients with symptomatic OCDs in the context of an otherwise healthy joint. These patients have often exceeded the ideal age for biological treatments but are still not eligible for TAR or AA. Another pool of patients consists of patients with a failed primary treatment.

These patients can be treated with an osteochondral graft or with a small metallic implant. The advantage of such a metallic implant is the absence of donor‐site morbidity [18].

More specifically, over the years, there has been a development of a small metallic implant to fill the defect, initially with HemiCAP Talus®, until the recent development of the second‐generation patient‐specific metal implant (Episealer® Talus Implant, Episurf Medical) designed based on CT and magnetic resonance imaging (MRI)images.

### PREOPERATIVE PLANNING

The Episealer Talus® Implant is an individualized implant, manufactured from cobalt chrome with a highly polished articular surface that precisely matches the geometry of the patient's talar OCD using an interpolating algorithm [18]. The design is composed of the hat, which aligns with the surface of the subchondral bone, and the press‐fit peg, made with titanium and hydroxyapatite to promote rapid and solid osteointegration. The Episealer Talus® implants are circular with diameters of 15 mm and variable depth.

The individualized design is based on MRI‐specific sequences and a dedicated CT protocol provided by the company to allow for a 3D computer reconstruction of the ankle articular surfaces. Patient data are tested for approval of image quality and allocated a patient‐specific code when uploaded to the in‐house web‐based platform [12]. After few days, with the mapping damage (Damage Marking Report [DMR]), the cartilage and bone structure of the entire ankle are assessed, particularly signs of osteoarthritis with joint space narrowing (Van Dijk classification's stage ≥2) are a contraindication as well as defects of the opposing tibial surface [19].

**Figure 1:**
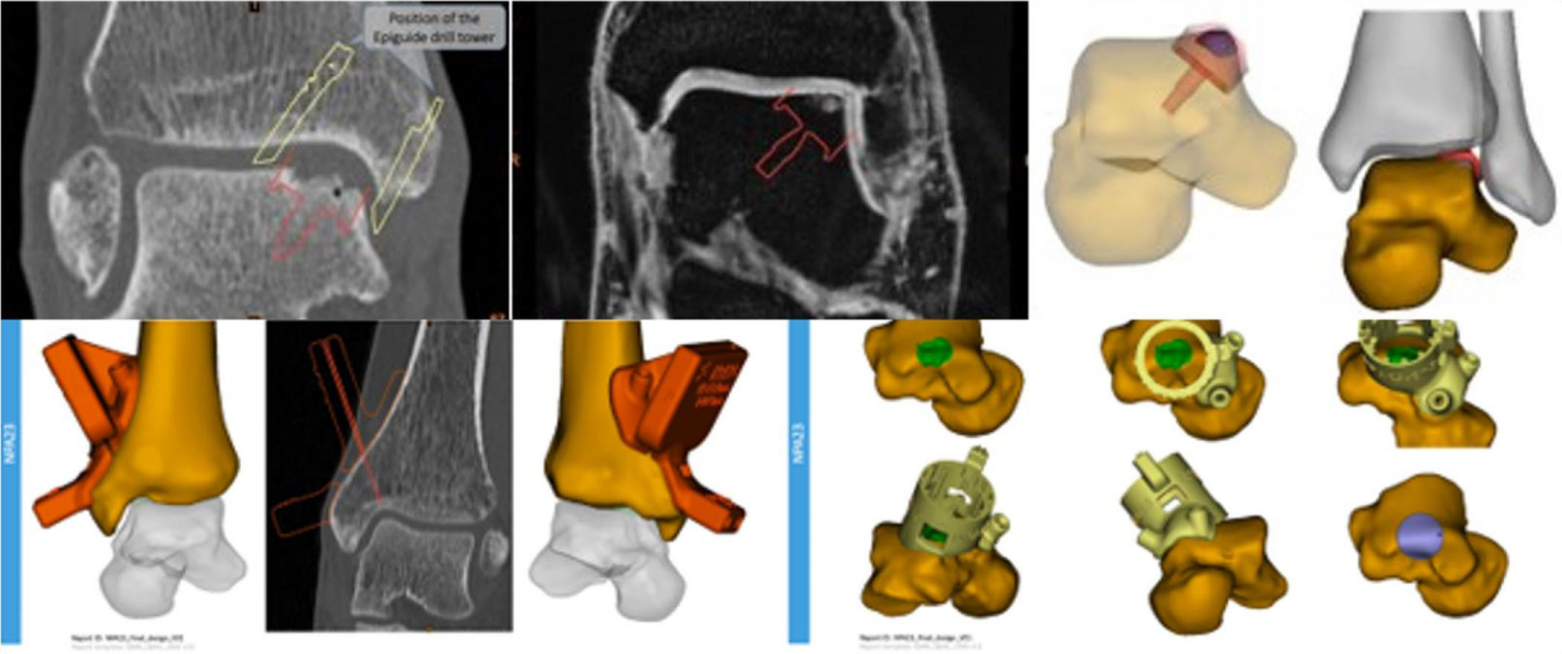
The Damage Marking Report based on computed tomography/magnetic resonance imaging scans (above) and the final design of the Damage Marking Report (below).

DMR is returned via the web platform to the surgeon for approval and, if necessary, the case is collectively discussed between surgeons and technicians possibly to perform positioning changes. Then, the final design is prepared, and the implant is sent into production and delivered to the hospital within approximately 5 weeks (Figure 1).

**Figure 2:**
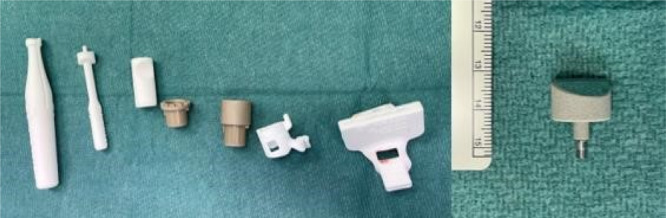
Custom‐made surgical tools set and definitive implant.

The definitive surgical tools set is composed of nine pieces; of those five are patient‐specific and 3D printed using polyamide (PA2200) (Figure 2).

## MEDIAL APPROACH

Under spinal or general anaesthesia, the patient is placed in the supine position, with feet at about 3 cm from the end of the operating table. A thigh tourniquet is applied and inflated for lower limb exsanguination, following the Association of Peri‐operative Registered Nurses (AORN) guidelines:

The tourniquet inflated intraoperatively to a pressure higher than the limb occlusion pressure. Intravenous antibiotic prophylaxis with a single dose of cephalosporin is administered 30 min prior to limb exsanguination. The affected leg and foot are prepared and draped.

A medial 4‐ to 5‐cm incision is performed along the medial malleolus. The tibial surface is carefully debrided both anteriorly and posteriorly, making sure not to damage any posterior tendons, neurovascular bundle and the periosteum. Medial capsulotomy of the ankle is performed and a Hohmann retractor is placed to protect all the anterior structures. The retinaculum flexor is incised in the retromalleolar zone to expose the posterior tibial tendon and to protect it with a Hohmann retractor, preparing the surface for the patient‐specific Medial Malleolus Osteotomy Guide (MMOG).

**Figure 3:**
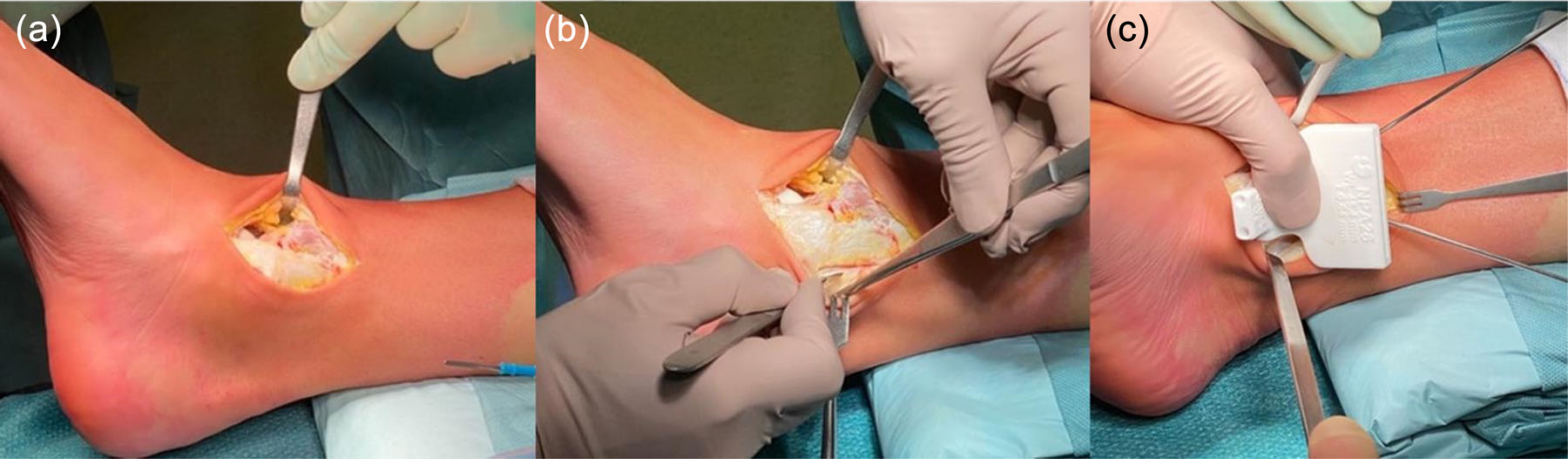
Ankle medial approach. (a) Capsulotomy of the ankle and positioning of a Hohmann retractor; (b) Incision of retinaculum flexor to expose tibialis posterior tendon; (c) Positioning of the Hohmann retractor to protect the tibialis posterior tendon and positioning of the Medial Malleolus Osteotomy Guide.

The MMOG is then positioned on the tibial surface verifying the proper anatomical fit (Figure 3).

Proper positioning of the osteotomy guide is crucial to allow the correct visualization of the OCDs and avoid iatrogenic lesions of the tibial cartilage surface, the medial ligamentous complex or Tibialis Posterior tendon which is protected with a Hohmann Retractor. The proximal holes of the MMOG are used for temporary fixation of the guide with two Kirschner wires, while distal ones are for fixation of the medial malleolus with two screws after the positioning of the implant.

**Figure 4:**
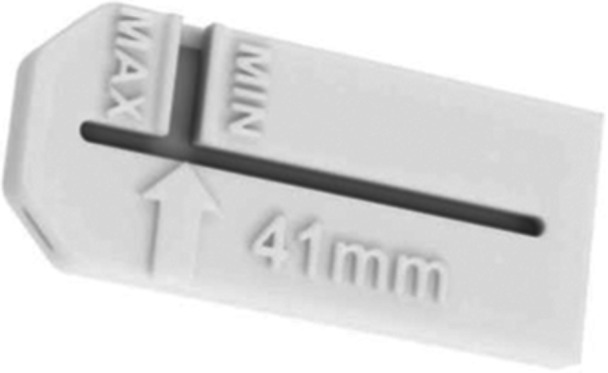
Checking guide (Osteotomy Depth Metre) for the right length of the saw.

The Osteotomy Depth Metre provides the possibility to check the correct length of the blade once mounted on the power tool; care must be taken because the assembly could determine a variation in the length of the blade (Figure 4). For this reason, it is recommended to empirically measure the final blade length after assembly during the first instance and communicate the result to the company.

Malleolar osteotomy is initiated and subsequently terminated by using an osteotome. Care must be taken with the osteotome to not change the cutting direction of the osteotomy, consequently creating bone ‘steps’ that can result in a medial slipping of the malleolus after the final osteosynthesis and consequent and consequent reduction of the medial gutter's space.

**Figure 5:**
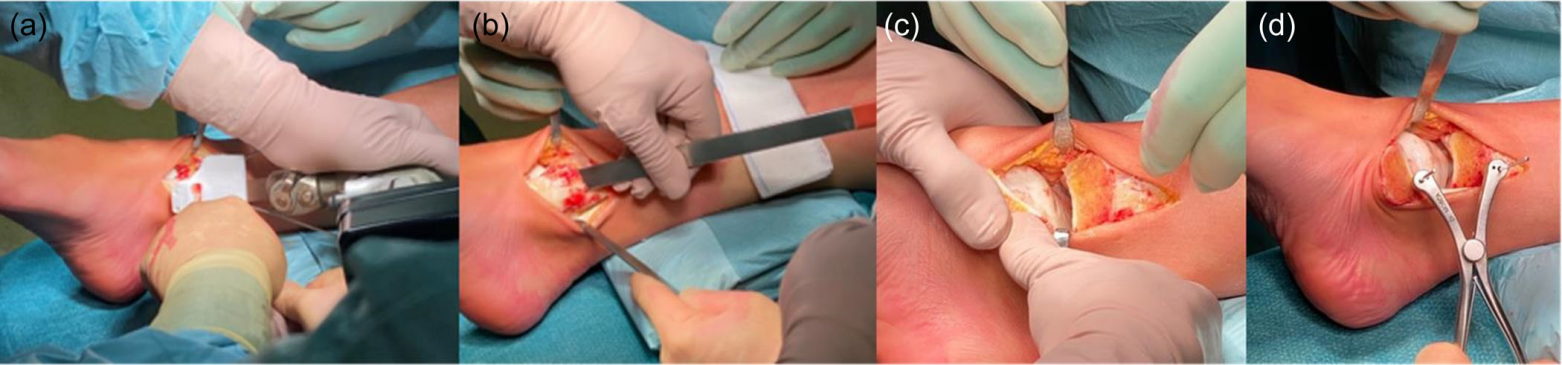
Medial exposure of the talar dome. (a) Malleolar osteotomy is initiated and (b) Subsequently terminated with an osteotome; (c) Overturning of the medial malleolus; (d) Increasing the articular space between tibia and talus using a Hintermann distraction forceps.

The medial malleolus is then overturned and temporarily fixed using Kirschner wires, leaving the talar dome and the osteochondral lesion now fully accessible. To facilitate this step, it may sometimes be necessary to perform a minimal release of the Deep Deltoid Ligament and posterior capsule when overturning the malleolus. To facilitate the placement of the Epiguide and to improve the talus direct visual exposure, distraction forceps (Hintermann spreader) can be used to separate the tibia and talus (Figure 5).

**Figure 6:**
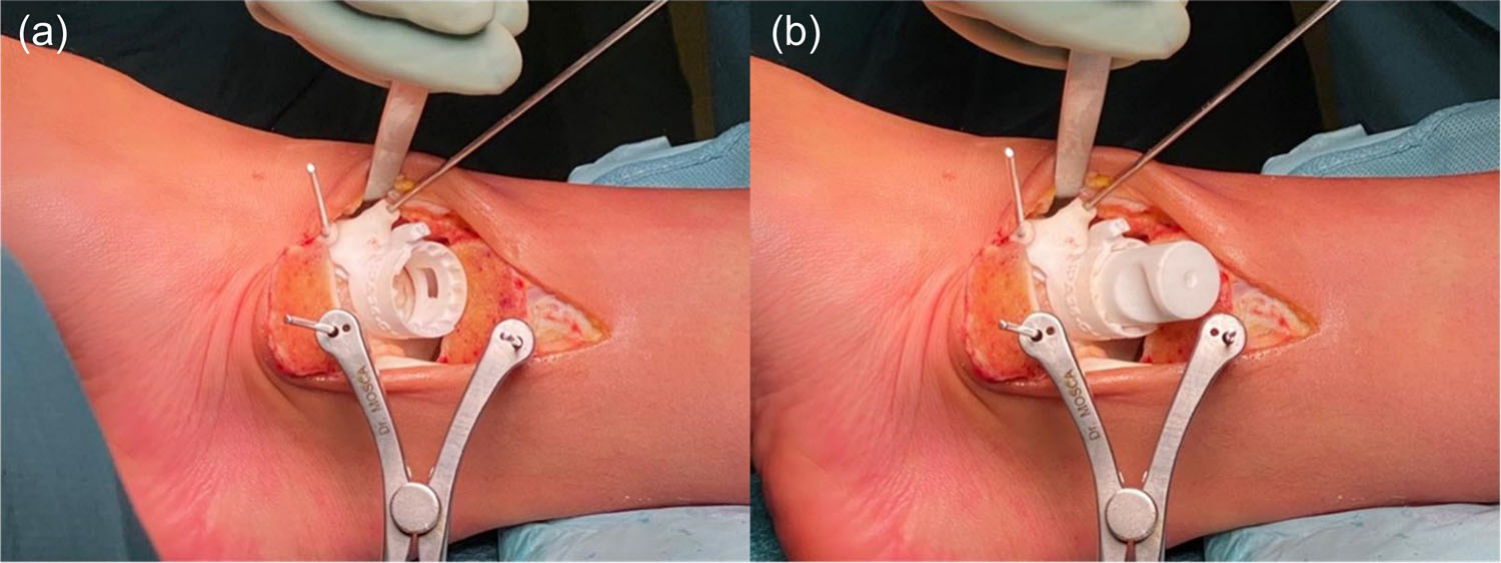
Identification of the osteochondral lesion. (a) Positioning of the Epiguide on the medial talar dome edge; (b) Insertion of the Pin Socket inside the Epiguide.

Subsequently, the Epiguide is carefully positioned on the articular cartilage edge of the talar dome, paying close attention to identifying its correct position with the help of the final renderings provided by Episurf. Two surgical pins are used to fasten the Epiguide to the bone, taking care of positioning it flush on the cartilage surface, without any gaps that could change the orientation of the next drilling resulting in an uneven sinking of the patient‐specific implant. The centre hole is created by introducing the Pin Socket inside the Epiguide and advancing a 2 mm Kirschner wire approximately up to about 15 mm, or otherwise up to the same depth of the final implant, taking care that it does not stop automatically on the Epiguide (Figure 6).

After that, the Drilling Socket is mounted in the correct position onto the Epiguide, to ensure alignment of the two arrows, one on the Drilling Socket and the other on the Epiguide, to avoid incorrect drilling. Then, the first milling is performed using Epidrill mounted on a power tool and drilled at full speed until the stop indicated by the edge of the Drilling Socket, ensuring the two components adhere well and avoid axial pressures. Since the Epiguide is stabilized with only two K‐wires anteriorly, at this point it may be necessary to further stabilize the Epiguide using the surgeon's fingers or surgical tools to further stabilize and avoid micromovements of the guide. Attention must be paid not to exert a force only in one direction to not misalign the Epiguide.

The final drill is performed by switching the Drilling Socket with the Adjustment Socket, allowing a fine adjustment of the depth. Ensure that the tip of the Epidrill is inserted into the predrilled hole and that the drill body is not in contact with the cartilage surface when the drilling procedure starts. Epidrill must be used applying only moderate force until the stop on the Adjustment Socket.

**Figure 7:**
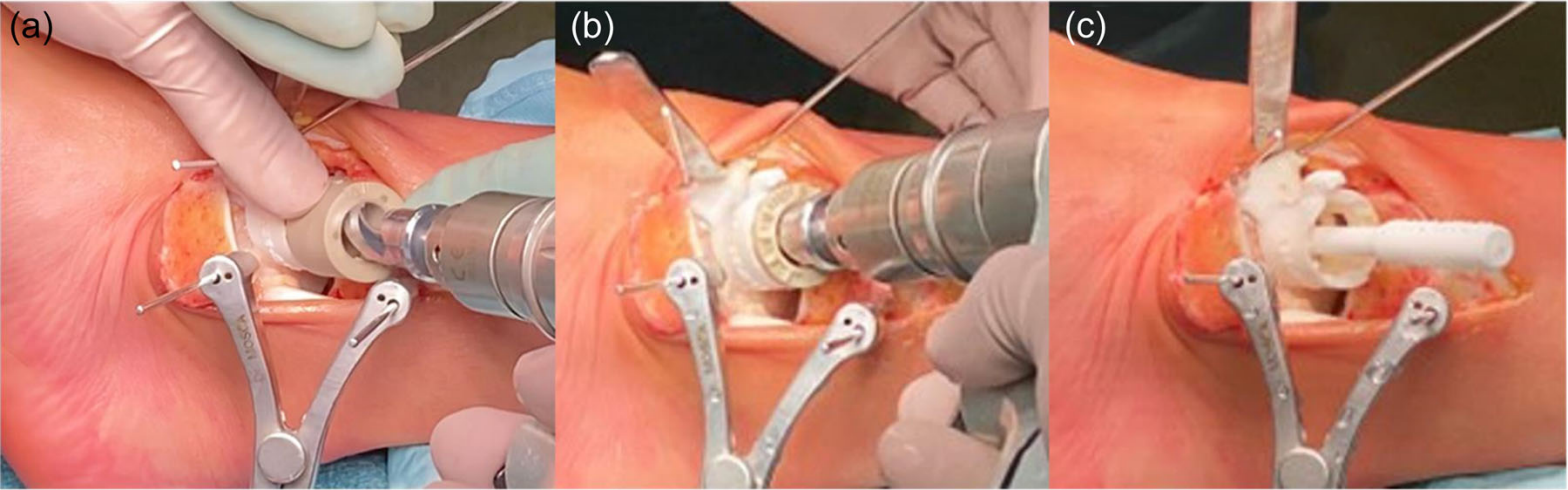
Creation of the implant housing. (a) Reaming with the Epidrill; (b) Final drill using the Adjustment Socket, for an accurate submillimetric adjustment of the depth; (c) Check of the implant recession by using the Epidummy, an exact replica of the shape of the final implant.

Vigorous washes through the whole drilling procedure will help reduce heat damage and eliminate cartilage fragments that may avoid osteointegration. Take care not to damage the cartilage edges of the defect. The depth of the final hole can be accommodated by rotating the Adjustment Socket and the drill depth can be incrementally increased in steps of 0.2 mm for each grade of rotation. The goal is to get the right depth with the least amount of redrilling. The depth of the hole is evaluated by using the Epidummy, which is an exact replica of the implant, and permits, once aligned with its rotation mark, to compare the depth of the Epidummy top surface with the surrounding cartilage edge and asses the recession of the implant (Figure 7). The goal is to place the Episealer Talus about 0.5 mm below the cartilage surface; if it is placed too superficially, a redrill is needed because it may damage the surrounding soft tissue. The correct measurement of the sinking can empirically be evaluated with a simple test: the surgeon's nail or an arthroscopic probe must be stopped during the sliding toward the ‘step’ with the healthy surrounding cartilage. If this happens, the final implant will not be too superficial. Sometimes due to asymmetrical reaming, the recession of the Epidummy is not correct, with one side more superficial and the other one deeper. In these cases, the defect can be manually corrected by applying stronger pressure in the more superficial portion during the next reaming.

**Figure 8:**
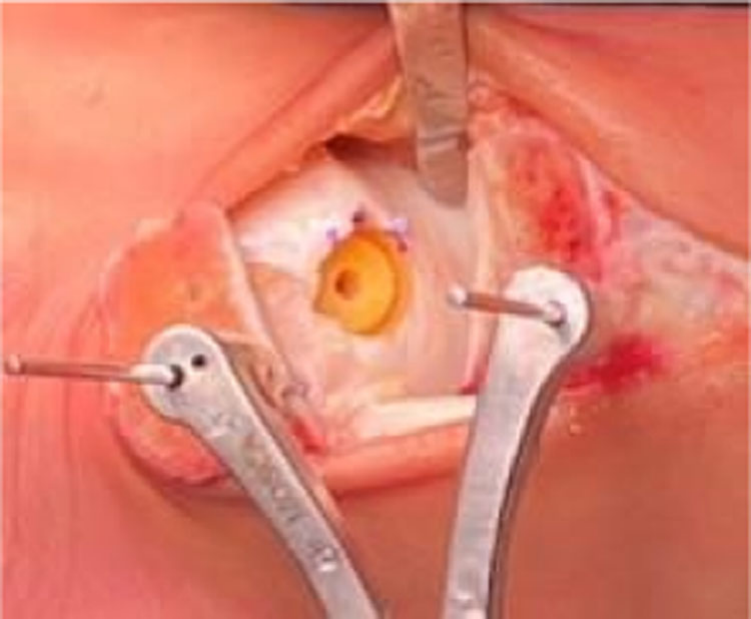
Definitive implant housing. Note the pen marks for the direction of rotation.

**Figure 9:**
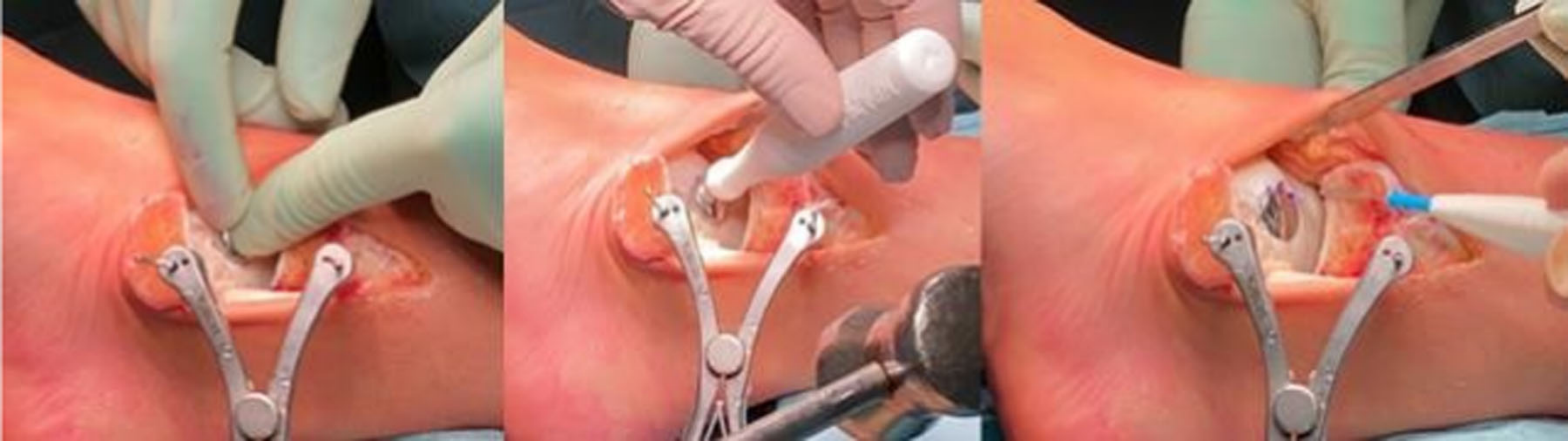
Placing the Episealer talus into the drilled hole.

Once a satisfactory result has been achieved, a sterile pen is used to mark the direction of rotation, and a new check of the depth is assessed with the Epidummy after the removing of the Epiguide (Figure 8). Finally, the Episealer Talus is placed into the drilled hole, paying attention to maintain the correct orientation and the alignment. It is advised to manually push the implant carefully into the hole, regularly checking the correct rotation. In case of change, it can still be adjusted by means of a Kocher forceps. The Epimandreal and a hammer are used to gently tap down the final implant until they reach the bottom of the bone hole providing a press‐fit fixation. Once fully inserted, the sound will become more distinct (Figure 9).

**Figure 10:**
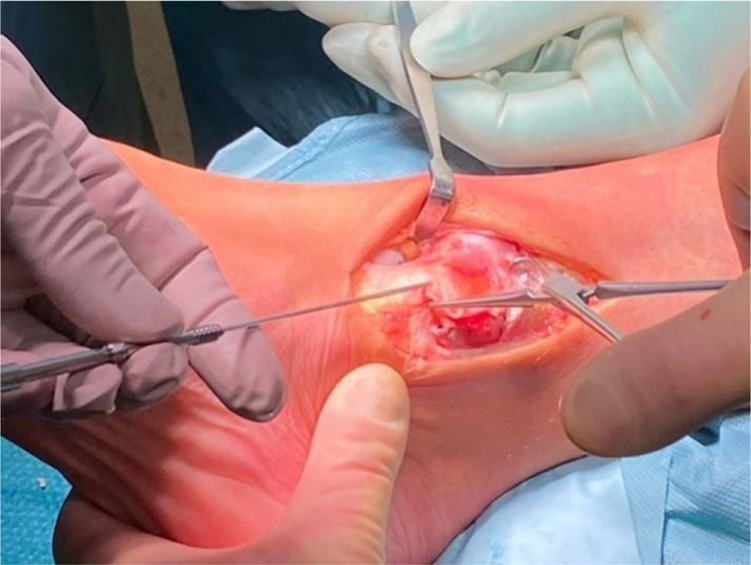
Anatomical reduction of the medial malleolus and osteosynthesis with two compression screws.

After the anatomical reduction of the medial malleolus using forceps reductors, the osteosynthesis is performed with two cancellous cannulated screw along the predrilled holes (Figure 10).

**Figure 11:**
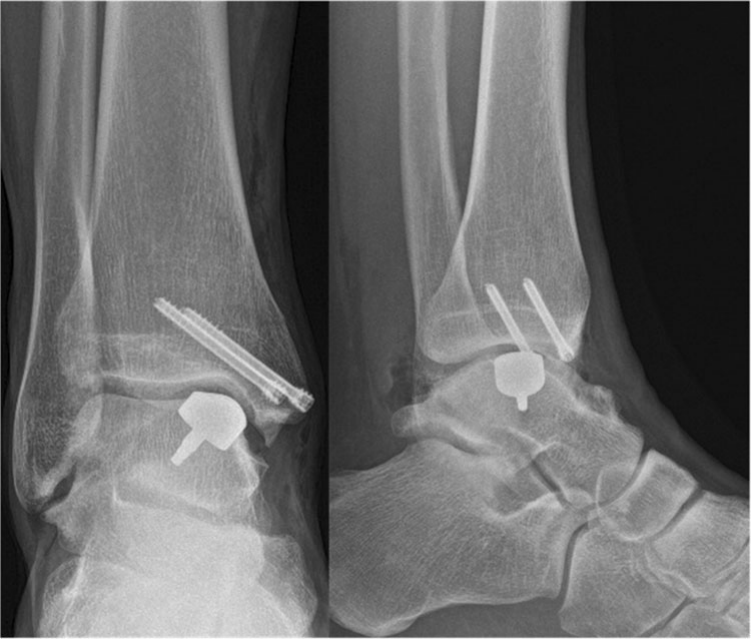
Postoperative x‐rays for medial osteochondral defect.

The tourniquet is deflated and tourniquet time recorded. Haemostasis is carefully performed, and flexor retinaculum of foot closed with few knots to avoid fibrosis and impingement with Tibialis Posterior tendon. Skin is closed, being careful of tension, with interrupted absorbable no. 3–0 suture. Check the postoperative x‐ray (Figure 11).

## LATERAL APPROACH

A lateral curved incision of 5 or 6 cm, overcrossing the tip of the lateral malleolus distally elongated to the sinus tarsi is performed. The connective tissues and the Extensor Digitorum muscle are carefully dissected. With the ankle in dorsiflexion, the fascia is incised to disclose the muscle belly to ensure no damage to any anterior tendons and neurovascular bundle. Lateral capsulotomy of the ankle is performed by removing a part of the joint capsule and articular fatty tissue.

**Figure 12:**
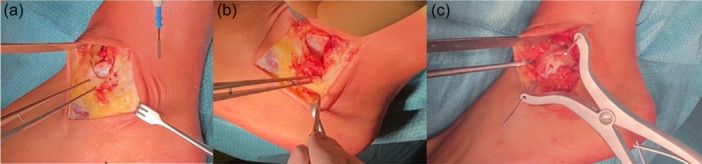
Ankle lateral approach. (a) Lateral capsulotomy of the ankle is performed by removing a part of the joint capsule; (b) Removing the anterior talofibular ligament + calcaneofibular ligament as ‘one flap of tissue’ and anterior dislocation of the talus with a Hintermann spreader.

Opening of the sheet of the peroneal tendons distal to the tip of the lateral malleolus is performed to expose the peroneal tendons and to protect them. Then, the release of the anterior talofibular ligament (ATFL) and the calcaneofibular ligament (CFL) ligaments is performed. Ensure preservation of the connection between ATFL and CFL. Remove the ATFL + CFL as ‘one flap of tissue’ from the fibula. The talus is now easily dislocated anteriorly to expose the lesion with the help of a Hintermann spreader (Figure 12).

**Figure 13:**
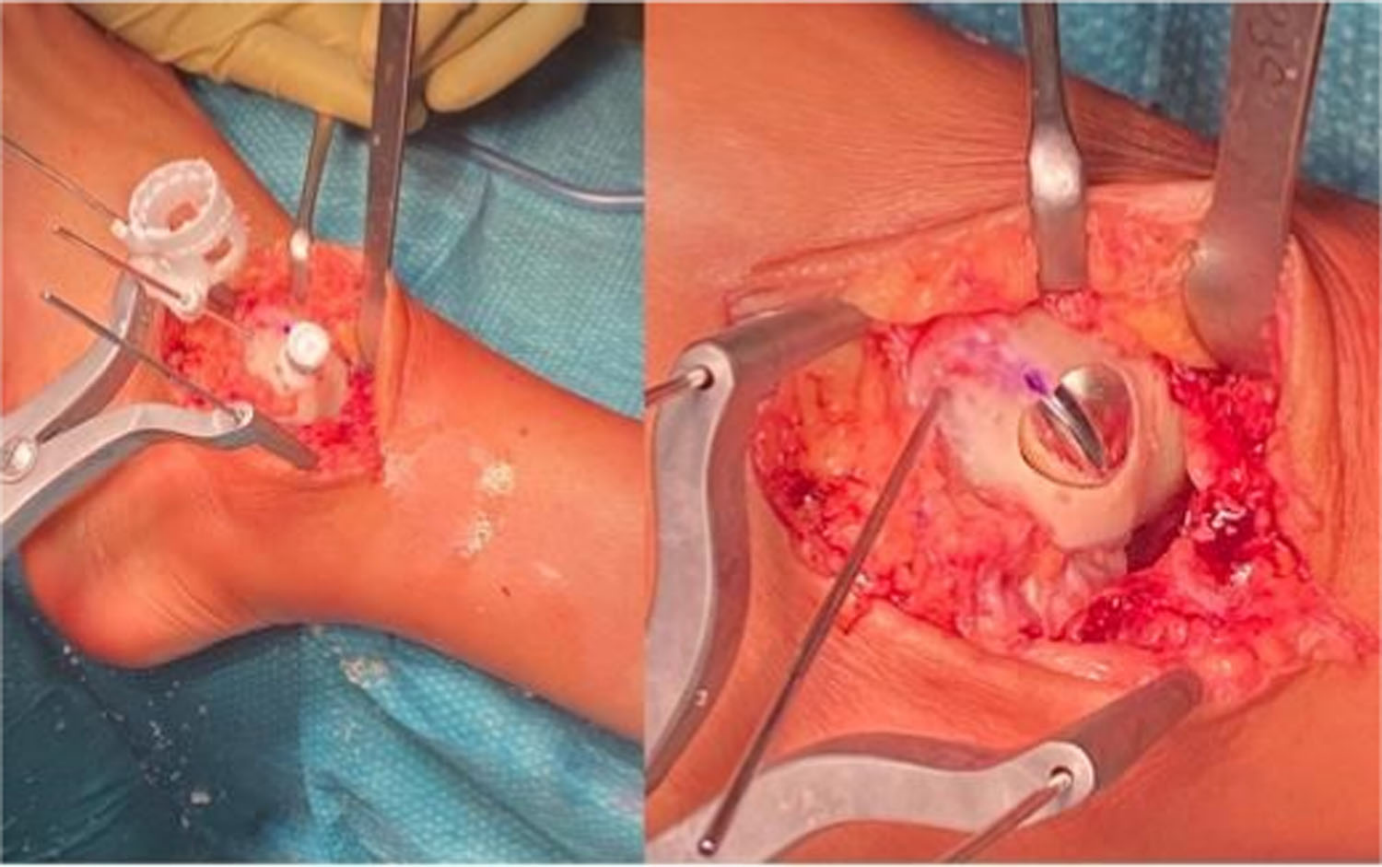
Implant of the temporary (on the left) and final (on the right) component.

The Episealer Talus is implanted according to the standard technique already described before (Figure 13).

**Figure 14:**
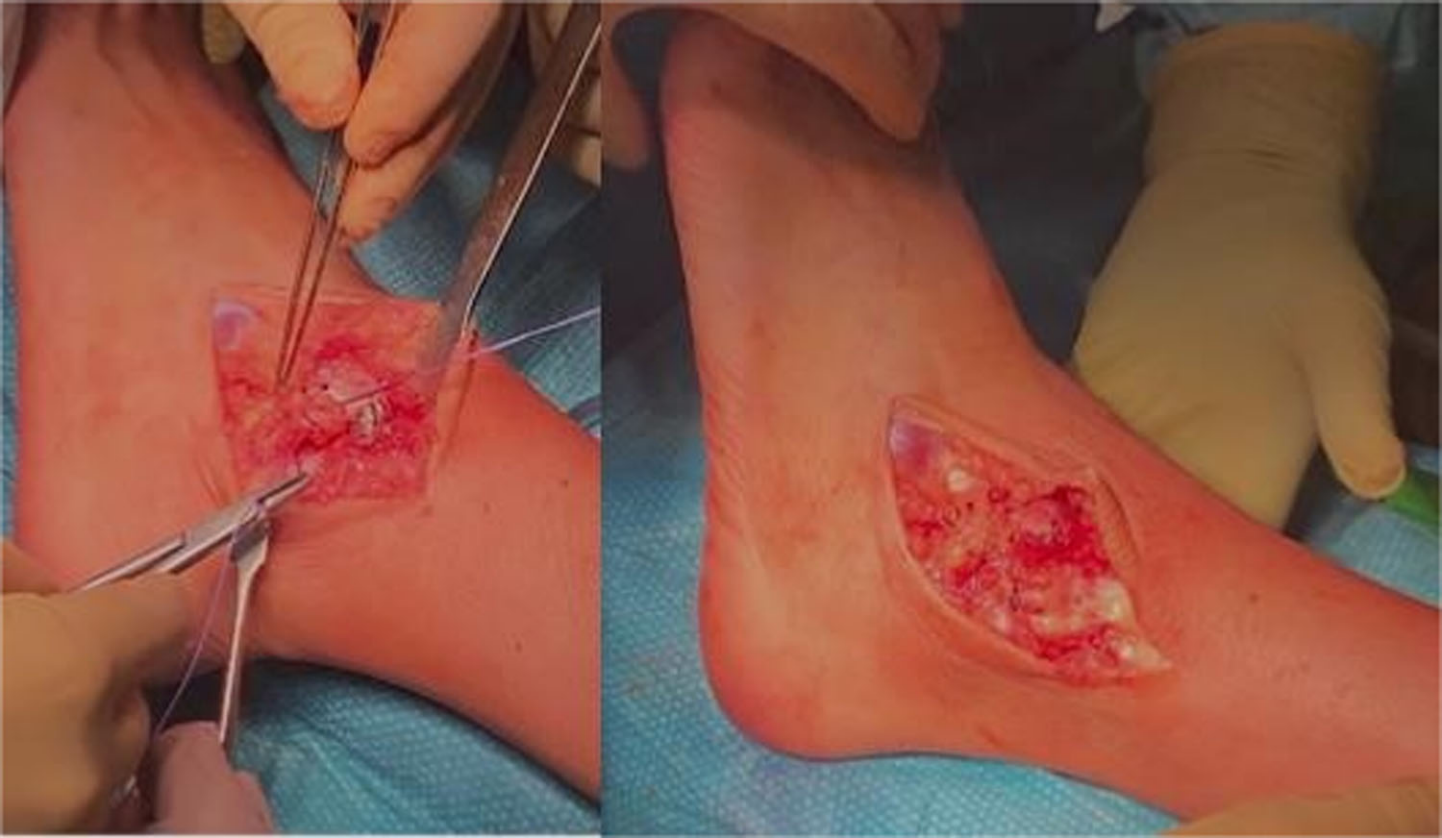
External capsule‐ligamentous reconstruction with a nonanatomical technique using Bunnell sutures.

**Figure 15:**
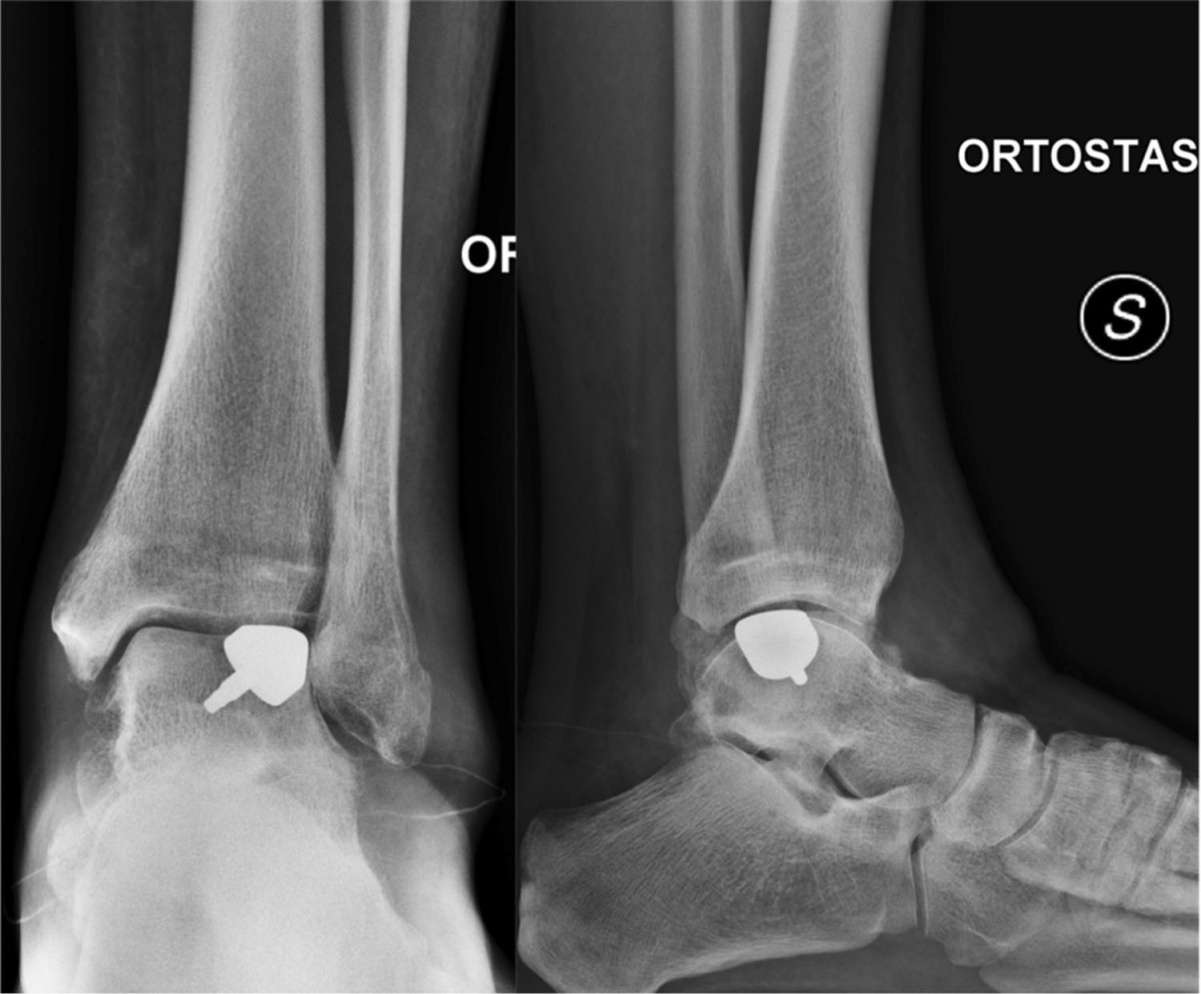
Postoperative x‐rays for lateral osteochondral defect.

Finally, an external capsule‐ligamentous reconstruction is performed with an anatomical technique using Bunnell sutures. Posterior incision, proximal to the peroneal tendon retinaculum, is performed; then, a raw bone surface is created on the anterior fibula tip and two 2.0 mm holes in the fibula tip are drilled. The sutures are pulled through using the suture passer and knot with the foot in everted position. Then, a rectangle‐shaped periosteum flap is carved, and the remaining sutures are crossed over the denuded fibula tip, to secure and achieve a tight reconstruction. Two transosseous sutures are made to ensure the tension of the peroneal tendon retinaculum. Alternatively, the ATFL and CFL can be fixed back to the fibula by using bone anchors. Skin is closed, being careful of tension, with interrupted absorbable no. 3–0 suture (Figure 14). Check the postoperative x‐ray (Figure 15).

### Additional procedures

Before Episealer Talus, ankle dorsiflexion capability is evaluated maintaining the hindfoot in correction with the knee in extension. If 90° cannot be obtained, an Achilles lengthening is performed. Procedures such as lateral and/or medial ligamentous reconstruction and subtalar arthrodesis may be necessary depending on the clinical evaluation to protect the implant and reduce the risk of revision. A lateral shift calcaneal osteotomy can be performed even when the pathology is not symptomatic to unload the medial talar dome in case of medial OCDs [22].

### Postoperative management

A nonweight‐bearing short‐leg cast to protect the operative sites is positioned for 6 weeks, immobilizing the ankle at approximately 90°. In the first 2 weeks after surgery, it is important to take care of the wound and detect early symptoms of skin suffering that could result in delayed wound healing. Active and passive mobilization of the ankle can be started 7 days after surgery.

Immediate postoperative and 6‐week postoperative anteroposterior and lateral x‐rays of the ankle are obtained. After 6 weeks, a check of the medial malleolus consolidation, progressively increasing weight‐bearing, initially with two crutches and the leg boot, is allowed. Subsequently, a specific physiotherapy programme is started to recover adequate range of motion and strength of the limb.

## DISCUSSION

OCD of the talus usually results from traumatic insult. Various procedures are available for the treatment of OCDs; however, there is still not enough evidence of what could be considered the best treatment [4,7,20,23].

The goal of operative treatment of talar OCDs with Episealer Talus is to achieve an improvement in symptoms and function maintaining as much mobility as possible in the foot and ankle articular complex. Moreover, in literature, the long‐term effects of mini‐metal implants seem to suggest a chondroprotective effect in adequately selected patients supporting the surrounding articular cartilage reducing the progression of defects [12].

In literature, long‐term clinical results for first‐generation metal talar implants are debated likely because the surgical technique can be challenging. It is not excluded that poor clinical results of some authors could depend on the positioning of the implant, indeed minimal positioning variation could not be tolerated in such a congruence joint and can damage surrounding tissues [3,5,8,22,14].

Possible advantages with second‐generation implants can be more precise approaches by means of a patient‐specific MMOG, more precise placement of the implant by means of a patient‐specific implant positioning guide and a patient‐specific implant that is tailor‐made to cover a medial or lateral talar OCD. However, to date, there are still no studies that compared the outcomes between first‐ and second‐generation implants.

Patients' eligibility for Episealer Talus also is crucial: the main indications for Episealer Talus implantation are active, middle‐aged patients (35–65 years old) with an OCD larger than 107 mm², involving the subchondral bone (subchondral cysts). These are patients falling into the ‘treatment gap’, who have large OCDs and a previous failure of the regenerative treatment or have passed of ideal age for biological treatment. This therapy can be considered the last attempt at joint‐preserving surgery in a symptomatic young patient before proceeding with TAR or AA, which could nonetheless be the final option.

To minimize the risk of intra/perioperative complications, there are a series of tips and tricks that can be considered.

Correct execution of the operative approach, proper positioning of the guides and the use of Hintermann spreader allow a perfect visualization of the OCDs minimizing the risk of iatrogenic lesions to the medial or lateral structures. Sometimes in the medial approach, a minimal release of the Deep Deltoid Ligament and posterior capsule, when overturning the malleolus, can facilitate the placement of the Epiguide.

The execution of the linear osteotomy of the malleolus is crucial to avoid bone steps that could determine a slip of the malleolus during the osteosynthesis and result in the reduction of the medial gutter's clear space. Proper triplanar alignment of the Epiguide, its strong stabilization during the positioning and the use of vigorous washes minimize the potential damage to healthy cartilage. Intraoperative washes also eliminate cartilage fragments that may avoid osteointegration.

Since the Epiguide is stabilized with only two K‐wires anteriorly, further stabilization of its posterior part with fingers or surgical tools (to avoid micromovements of the guide) can reduce the risk of an asymmetric implant recession.

The surgical positioning of the implant assisted by the intraoperative use of DMR and the conformity with surrounding articular cartilage to avoid high pressure is crucial. Implants should not be inserted such that the hat protrudes leading to potential damage on the tibial articular surface and persisting pain. The goal is to place the Episealer Talus about 0.5 mm below the cartilage surface; if it is placed too superficial, a redrill is needed because it may damage the opposing cartilage. The correct measurement of the sinking can empirically be evaluated with a simple test, the surgeon's nail or an arthroscopic probe must be stopped during the sliding toward the ‘step’ with the healthy surrounding cartilage. If this happens, the final implant will not be too superficial. Sometimes due to asymmetrical reaming, the recession of the Epidummy is not correct, with one side more superficial and the other one deeper. In these cases, the defect can be manually corrected by applying stronger pressure in the more superficial portion during the next reaming.

Before implanting Episealer Talus, if the preoperative MRI shows a large subchondral cyst that would not be filled by the implant at all, then the cancellous bone produced by the reaming can be used to fill the cyst and increase the osteointegration and stability of the implant. Exacting instrumentation, detailed MRI evaluation and surgical experience address these issues. Correct preoperative planning is crucial.

In the case of lateral OCDs, an external capsule‐ligamentous reconstruction is performed with an anatomical technique using Bunnell sutures as described previously.

In the first 2 weeks after surgery, it is important to take care of the wound and to detect early symptoms of skin suffering that could result in delayed wound healing and extend postoperative recovery times.

Surgical experience is needed to consider all the factors influencing lower limb alignment and bone, soft tissue or ligament balancing. Sometimes additional procedures can be necessary even if the defects are not symptomatic to protect the implant and reduce the risk of revision, like lateral shift calcaneal osteotomy to unload the medial talar dome in case of medial OCDs [22].

## CONCLUSION

Episealer talus for talar OCDs represents an additional tool for surgeons and patients. It is important to consider the correct indication and avoid mistakes in implant placement and malleoli osteosynthesis. This second‐generation resurfacing implant has multiple advantages which include a precise approach by means of a patient‐specific MMOG, a precise placement of the implant by means of a patient‐specific implant positioning guide and a patient‐specific implant that is tailor‐made to cover a medial or lateral talar OCD.

## AUTHOR CONTRIBUTIONS

All authors confirm that the article has never been published and is not under consideration for publication elsewhere. The paper has not been submitted to any other journal. All the authors meet the criteria for authorship. All the authors have read and approved the final manuscript and the data being presented in the manuscript, and are fully responsible for it.

## CONFLICT OF INTEREST STATEMENT

The authors declare no conflict of interest.

## ETHICS STATEMENT

All patients were informed about the surgical procedure, postoperative treatment and possible complications and signed a written informed consent. The study was conducted according to the Declaration of Helsinki.
